# MicroRNA-491-5p suppresses cervical cancer cell growth by targeting hTERT

**DOI:** 10.3892/or.2021.8199

**Published:** 2021-10-04

**Authors:** Qiang Zhao, Ying-Xian Zhai, Huan-Qiu Liu, Ying-Ai Shi, Xin-Bai Li

Oncol Rep 34: 979-986, 2015; DOI: 10.3892/or.2015.4013

Following the publication of this paper, an interested reader drew to the attention of the Office that the GAPDH control bands shown in Fig. 5 were strikingly similar to data appearing in different form in other articles by different authors. Owing to the fact that the contentious data in the above article had already been published elsewhere, or were already under consideration for publication, prior to its submission to *Oncology Reports*, the Editor has decided that this paper should be retracted from the Journal. The authors independently contacted the Editorial Office requesting that the paper be retracted.

The Editor apologizes to the readership for any inconvenience caused.

